# Liposomal Bupivacaine in Single‐Injection Quadratus Lumborum Block for Pediatric Kidney Transplant: Case Report of a Novel Application

**DOI:** 10.1111/petr.70112

**Published:** 2025-06-01

**Authors:** Rebecca Bonaroti, Armando Ganoza, Mihaela Visoiu

**Affiliations:** ^1^ Department of Anesthesiology and Perioperative Medicine University of Pittsburgh Medical Center Pittsburgh, PA USA; ^2^ Department of Pediatric Transplant Surgery UPMC Children's Hospital of Pittsburgh Pittsburgh, PA USA; ^3^ Department of Pediatric Anesthesiology UPMC Children's Hospital of Pittsburgh Pittsburgh, PA USA

**Keywords:** case report, kidney transplant, liposomal bupivacaine, pediatric, quadratus lumborum nerve block

## Abstract

**Background:**

Liposomal bupivacaine is FDA‐approved long‐acting bupivacaine for intraoperative local wound infiltration in pediatric patients 6 years and older, but not for ultrasound‐guided peripheral nerve blocks. There are few prior reports of using liposomal bupivacaine for preoperative peripheral nerve blocks in pediatric patients. We report the first use of liposomal bupivacaine in a single‐injection quadratus lumborum block in a pediatric patient undergoing a kidney transplant.

**Methods:**

Our patient is a 16‐year‐old female undergoing a living‐related kidney transplant for end‐stage renal disease. Pertinent past medical history includes symptoms of opioid withdrawal during a prior hospitalization. To optimize postoperative pain control, a preoperative single injection quadratus lumborum nerve block was performed with liposomal bupivacaine, and opioids and acetaminophen were administered as needed in the perioperative period.

**Results:**

The patient had excellent pain control for the first 36 h postoperatively and adequate pain control for the entire hospital stay, with minimal opioid administration. She reported decreased sensation to temperature and touch over her entire ipsilateral abdomen/flank for the first 48 h. Postoperative day 4 marked her highest average pain score, coinciding with the reported 72 h of efficacy of liposomal bupivacaine. She was able to ambulate less than 24 h postoperatively, did not report any withdrawal symptoms, and had no adverse events.

**Conclusions:**

This is the first documented case of liposomal bupivacaine in a preoperative quadratus lumborum nerve block for a pediatric patient. More studies are required to investigate the pharmacokinetics of liposomal bupivacaine in kidney recipients and determine its efficacy and safety.

**Abbreviations**
LAST: local anesthetic systemic toxicity
PCA: patient‐controlled analgesia
POD: postoperative day
QLM: quadratus lumborum muscle
QLs: quadratus lumborum nerve blocks
RCT: randomized controlled trial
TLF: thoracolumbar fascia

## 1 Introduction

Quadratus lumborum nerve blocks (QLs) with bupivacaine are part of a multimodal approach to postoperative pain management in pediatric kidney transplantation. While these blocks provide adequate pain relief, the duration of postoperative analgesia is often short. Liposomal bupivacaine is an FDA‐approved long‐acting local anesthetic for adult peripheral nerve blocks. In pediatric patients, it is only approved for use in intraoperative local wound infiltration [1]. The liposomal form of bupivacaine has higher lipid solubility and extensive protein‐binding properties, which enhance its potency and prolong the duration of peripheral nerve blockade [2]. Unlike standard bupivacaine, whose plasma levels decrease sharply within 3–6 h and whose clinical effects wear off within 24 h, the liposomal formulation of bupivacaine releases the drug gradually, providing analgesia for up to 72 h as documented in cases of intraoperative wound infiltration [2, 3].

There are few reports on using liposomal bupivacaine in wound infiltration in pediatric patients. These studies have demonstrated the safety of this method of administration, but there is no clear evidence that it provides superior analgesic benefits compared to standard local anesthetics [4, 5]. We found only one study comparing liposomal versus plain bupivacaine in preoperative adductor canal nerve blocks for adolescents undergoing orthopedic surgeries [6]. Additionally, there is a documented case of liposomal bupivacaine used postoperatively in femoral and sciatic nerve blocks for a pediatric patient who underwent a traumatic amputation [7]. However, there are no reports of its use in a quadratus lumborum block. This case report aims to document the first use of liposomal bupivacaine in a preoperative quadratus lumborum nerve block for a pediatric patient undergoing a kidney transplant. A written informed consent for publication was obtained from the patient and family.

## 2 Patient and Methods

Our patient was a 16‐year‐old female (43 kg, 153 cm, BMI 18.3, ASA 3) undergoing a kidney transplant for end‐stage renal disease secondary to p‐ANCA glomerulonephritis. She had a history of opioid withdrawal symptoms during a previous extended stay in the intensive care unit while attempting to taper off opioid analgesics. As a result, the patient, her family, and the care team aimed to minimize the risk of prolonged opioid use after surgery during this admission. Special approval was obtained from the patient and her family, the pharmacy, and the transplant surgeon to use liposomal bupivacaine off‐label for a peripheral nerve block in this pediatric patient.

**FIGURE 1:**
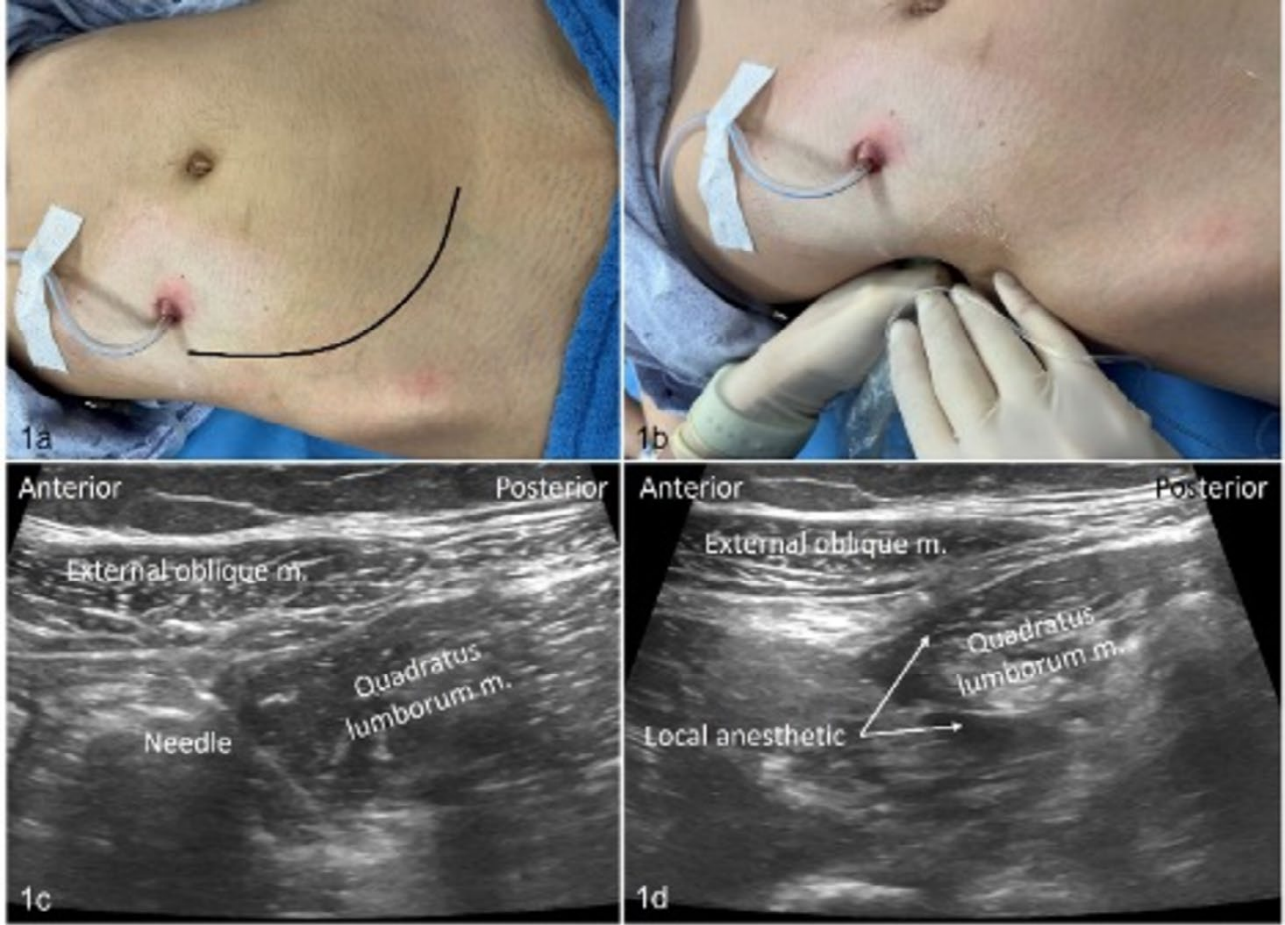
Quadratus lumborum block technique. (a) Incision location, (b) ultrasound probe and needle orientation, (c) ultrasound image of quadratus lumborum muscle and needle orientation, and (d) local anesthetic spread around the lateral and anterior borders of the quadratus lumborum muscle.

An ultrasound‐guided unilateral quadratus lumborum nerve block, single injection using an anterior approach, was performed preoperatively after general anesthesia was induced [8]. The patient was positioned supine with a slight lateral tilt by placing a towel roll under the posterior lumbar region (see Figure 1a). Under aseptic conditions, a high‐frequency probe (12 L, 55 mm linear broadband array) connected to a GE Healthcare ultrasound machine (GE Medical Systems, 9900 Innovation Drive, Wauwatosa, WI 53226, USA) was positioned transversely between the iliac crest and the costal margin (see Figure 1b *)*.

After identifying the three abdominal muscles—the external oblique, internal oblique, and transversus abdominis—the probe was moved posteriorly to visualize the quadratus lumborum muscle (QLM). A 22‐gauge, 80 mm SonoTAP needle (PAJUNK, Medical Systems, L.P.) was then inserted in‐plane at the lateral edge of the probe and advanced transmuscularly from anterior to posterior, until the tip was positioned between the anterior lamina of the thoracolumbar fascia (TLF) and the anterior border of the QLM (see Figure 1c). Correct needle placement was confirmed by injecting 2 mL of normal saline, which spread anteroposteriorly beneath the QLM following negative aspiration. Once confirmed, the local anesthetic was administered, and its spread toward the psoas muscle was observed (see Figure 1d).

The medication administered in the nerve block consisted of 10 mL (133 mg, 3 mg/kg) of 1.3% liposomal bupivacaine and 5 mL of 0.25% plain bupivacaine (12.5 mg, 0.28 mg/kg). The total intraoperative analgesics administered intravenously included 100 mcg of fentanyl, 0.6 mg of hydromorphone, and 500 mg of intravenous acetaminophen. The patient was successfully extubated at the end of the operation and transferred to the pediatric intensive care unit. The patient was closely monitored for signs of local anesthetic systemic toxicity (LAST) following the block, including continuous observation of vital signs and neurologic status during the postoperative period. She was provided with a hydromorphone patient‐controlled analgesia (PCA) pump for postoperative pain management.

## 3 Results

The patient had excellent pain control for the first 36 h postoperatively. Pain scores were documented at rest or with ambulation every 2–4 h using self‐reported pain scores from 0 to 10 (Numeric Rating Scale, where 0 indicates no pain and 10 represents the worst possible pain). Her daily average, minimal and maximum pain scores, and analgesic use are presented in Table 1. On postoperative days (POD) 1 and 2, the patient experienced decreased sensation to temperature and touch over the ipsilateral abdomen and flank, corresponding to the area anesthetized by the preoperative quadratus lumborum nerve block. She had numbness from T10 to L1 dermatomes through POD 1, then from T10 to L2 on POD 2. She was able to ambulate less than 24 h postoperatively. On POD 2, she was transferred from the pediatric intensive care unit to the regular floor, at which point she was also transitioned from a hydromorphone PCA to as needed oral oxycodone 5 mg tablets. She no longer required breakthrough intravenous analgesics after POD 4. After reaching the highest self‐reported pain scores on POD 4, pain levels significantly decreased from POD 5 to 8. The patient was discharged home on POD 8 with a prescription for ten 5 mg oxycodone tablets.

**TABLE 1:** Daily average, minimum, and maximum pain scores, and analgesic administration. Pain scores by self‐reported Numeric Rating Scale of 0 (no pain) to 10 (worst imaginable pain).

Postoperative day	Daily average pain score (0–10)	Minimum pain score (0–10)	Maximum pain score (0–10)	Total daily analgesic medications
POD 1	2.4	0	6	Acetaminophen 60 mg/kg
				Hydromorphone 0.06 mg/kg
POD 2	5.0	0	7	Acetaminophen 60 mg/kg
				Hydromorphone 0.03 mg/kg
				Oxycodone 0.49 mg/kg
POD 3	5.2	2	7	Acetaminophen 60 mg/kg
				Hydromorphone 0.01 mg/kg
				Oxycodone 0.49 mg/kg
POD 4	5.5	2	8	Acetaminophen 45 mg/kg
				Hydromorphone 0.005 mg/kg
				Oxycodone 0.12 mg/kg
POD 5	1.7	0	5	Acetaminophen 60 mg/kg
				Oxycodone 0.23 mg/kg
POD 6	2.0	0	5	Acetaminophen 15 mg/kg
				Oxycodone 0.23 mg/kg
POD 7	2.8	0	7	Acetaminophen 60 mg/kg
				Oxycodone 0.23 mg/kg
POD 8	3.0	0	7	Oxycodone 0.12 mg/kg

## 4 Discussion

Adequate pain control is crucial for the well‐being and comfort of pediatric kidney recipients. It reduces suffering and helps them recover faster and participate in postoperative activities. Managing postoperative pain effectively is challenging, with intravenous opioids being the most common analgesics used. For pediatric kidney transplant recipients, minimizing opioid exposure is particularly important due to the risks associated with long‐term opioid use, such as graft loss and increased mortality [9]. Factors such as coagulopathy during surgery and the need for anticoagulation increase the risk of bleeding at injection sites with peripheral or neuraxial nerve block catheters. Therefore, single‐injection nerve blocks are often preferred for pediatric transplant patients.

For patients with a history of opioid withdrawal symptoms, it is crucial to choose a pain management strategy that minimizes or eliminates the need for opioid medications. Hernandez et al. conducted a retrospective pediatric study on using quadratus lumborum nerve blocks (QLs) with ropivacaine for perioperative pain management in 32 children undergoing kidney transplants [8]. While these blocks provided effective pain relief, their duration was short, necessitating additional opioid administration. Liposomal bupivacaine has proven effective in adult peripheral nerve blocks, but its use in pediatric cases is limited. Most pediatric studies focus on local wound infiltration during surgery. A 2018 retrospective analysis of pediatric spine surgeries found no significant difference in postoperative pain or analgesic requirements for patients receiving liposomal bupivacaine [5]. A 2021 study by Tirotta et al. demonstrated that liposomal bupivacaine has an adequate safety profile when used for local wound infiltration in pediatric spine and cardiac surgeries, with no severe treatment‐related adverse events [4]. Additionally, Visoiu et al. showed that liposomal bupivacaine was more effective than plain ropivacaine in intercostal nerve blocks performed to treat chronic chest pain [10]. The established safety profile and similar side effects to plain bupivacaine made liposomal bupivacaine an effective choice for this case. Interestingly, a recent meta‐analysis, which included a study on the lateral QL nerve block, found no clinical advantage of liposomal bupivacaine over plain bupivacaine in abdominal fascial plane blocks [11]. The expected benefit of liposomal bupivacaine's prolonged analgesic effect may be reduced or confounded by clinical variables such as the type of surgical procedure, patient characteristics, variations in block technique, and the concurrent use of multimodal analgesia. Despite this, our patient had adequate pain control throughout the hospital stay. Opioid use in our patient remained low throughout her stay, and we did not observe an increase in postoperative opioid consumption on POD 1 once the effects of the plain bupivacaine wore off. The patient and family reported high satisfaction with the analgesic plan, noting effective pain relief and minimal discomfort throughout the postoperative period.

Postoperative pain typically worsens initially and improves over time. One key benefit of using liposomal bupivacaine in single‐injection nerve blocks is its ability to provide long‐lasting pain relief. In our case, the patient's highest pain score occurred on postoperative day (POD) 4, aligning with the reported 72‐h efficacy of liposomal bupivacaine [3]. This is consistent with the bimodal kinetics of the liposomal formulation, which provides prolonged pain relief compared to standard bupivacaine, whose plasma levels decline sharply after 3–6 h [3]. It is important to note that the pain scores reported by the patient were consistent with the sensory exam, which demonstrated the efficacy and duration of the block. This correlation further supports the effectiveness of the pain management strategy and the duration of analgesia the block provides.

In our case, the dosage of liposomal bupivacaine was guided by the FDA prescribing information for interscalene brachial plexus nerve blocks in adults, as no specific guidelines exist for pediatric peripheral nerve blocks [1]. To expedite the onset of the nerve block, plain bupivacaine was co‐administered with liposomal bupivacaine. A meta‐analysis by Bergese et al. found that higher doses of liposomal bupivacaine in wound infiltration led to a longer duration of postoperative pain control than lower doses [12]. This suggests that increasing the amount of liposomal bupivacaine in QL blocks could help extend the duration of pain relief. Liposomal bupivacaine suggests a safer profile with lower peak plasma levels and thus a lower risk of toxicity, especially in patients experiencing renal failure. On the other hand, safety margins in these children are reduced due to decreased excretion and decreased protein binding, which may lead to increased peak plasma levels. Monitoring bupivacaine levels could help correlate analgesia duration with the presence of numbness and bupivacaine blood concentration.

For patients with a history of opioid withdrawal symptoms, avoiding opioids is essential to prevent triggering withdrawal episodes and the associated physical and psychological discomfort. In our case, we successfully minimized opioid exposure, which improved both the patient's and family's satisfaction with the pain management approach. This was especially important because the patient had previously experienced opioid withdrawal symptoms during a prior admission, necessitating a slow taper of opioid analgesics—an experience she and her family wished to avoid repeating. By reducing opioid use in this case, we were able to alleviate anxiety regarding postoperative pain management and foster trust between the patient, family, and medical providers.

## 5 Conclusion

A quadratus lumborum block with liposomal bupivacaine provided effective and sustained pain relief for our pediatric patient undergoing kidney transplant. It reduced the need for opioid administration in the postoperative period, which positively impacted the patient's overall pain control experience. Further studies need to be performed to better understand the efficacy and safety of liposomal bupivacaine in kidney transplant recipients receiving peripheral nerve blocks.

## Data Availability

The data that support the findings of this study are available from the corresponding author upon reasonable request.
